# Probing feedforward and feedback contributions to awareness with visual masking and transcranial magnetic stimulation

**DOI:** 10.3389/fpsyg.2014.01173

**Published:** 2014-10-21

**Authors:** Evelina Tapia, Diane M. Beck

**Affiliations:** ^1^Beckman Institute, University of Illinois Urbana-ChampaignUrbana, IL USA; ^2^Department of Psychology, University of Illinois Urbana-ChampaignUrbana, IL, USA

**Keywords:** TMS, visual masking, awareness, feedforward, feedback, alpha oscillations, alpha phase, alpha power

## Abstract

A number of influential theories posit that visual awareness relies not only on the initial, stimulus-driven (i.e., feedforward) sweep of activation but also on recurrent feedback activity within and between brain regions. These theories of awareness draw heavily on data from masking paradigms in which visibility of one stimulus is reduced due to the presence of another stimulus. More recently transcranial magnetic stimulation (TMS) has been used to study the temporal dynamics of visual awareness. TMS over occipital cortex affects performance on visual tasks at distinct time points and in a manner that is comparable to visual masking. We draw parallels between these two methods and examine evidence for the neural mechanisms by which visual masking and TMS suppress stimulus visibility. Specifically, both methods have been proposed to affect feedforward as well as feedback signals when applied at distinct time windows relative to stimulus onset and as a result modify visual awareness. Most recent empirical evidence, moreover, suggests that while visual masking and TMS impact stimulus visibility comparably, the processes these methods affect may not be as similar as previously thought. In addition to reviewing both masking and TMS studies that examine feedforward and feedback processes in vision, we raise questions to guide future studies and further probe the necessary conditions for visual awareness.

## INTRODUCTION

The exact mechanism by which visual awareness arises and the neural circuits involved in generating this experience are greatly debated (Crick and Koch, 1995; Zeki and Bartels, 1998; Lamme, 2000, 2006a; Dehaene and Naccache, 2001; Dennett, 2001; Rees et al., 2002; Cooney and Gazzaniga, 2003; Crick and Koch, 2003; Lamme, 2003; Tong, 2003; Block, 2005; Lamme, 2006a; Zeki, 2008). However, many theories assume that some version of recurrent activity or feedback is necessary for awareness. Here we review findings from visual masking and transcranial magnetic stimulation (TMS) studies of visual suppression. In both paradigms visual awareness of a stimulus is disrupted at distinct points in time from the onset of the stimulus, and thus they have been used to elucidate the relationship between feedforward and feedback processes in awareness. The overlap in the windows of visual suppression with these two methods has been proposed to reflect a similar mechanism by which stimulus visibility is impaired (Breitmeyer et al., 2004a). That is, forward visual masking and TMS applied before the onset of a stimulus have been suggested to reflect interference with the feedforward visual processes; backward visual masking and TMS applied after the onset of the stimulus have been implicated in interference with the feedback visual processes. A closer examination of more recent studies, however, suggests that TMS effects that have been previously attributed to disruption of feedback processes may also reflect interference with feedforward visual processing. Additionally, there is increasing evidence that TMS can modulate alpha frequency oscillations that in turn can impact visual awareness. Such findings complicate the interpretation of TMS effects on awareness. For example, the documented influences of pre-stimulus alpha oscillations on awareness raise the possibility that the visual masks or TMS stimulation that precede the target may impact awareness by changing the brain state prior to the onset of the target. We argue that despite the current lack of clarity regarding their neural mechanisms, both visual masking and TMS are useful methods for studying the neural conditions necessary for visual awareness.

## THE VISUAL SYSTEM AND THE FEEDFORWARD-FEEDBACK FRAMEWORK

The visual system has abundant anatomical feedforward and feedback connections that are organized in a hierarchical manner (DeYoe and Van Essen, 1988; Felleman and Van Essen, 1991; Van Essen et al., 1992; Merigan and Maunsell, 1993; Felleman et al., 1997; Lamme and Roelfsema, 2000; Bullier, 2001). Initially, information enters the cortex in a feedforward manner. Electrophysiological studies in humans indicate that the visual signal from the retina reaches the primary visual cortex, V1, in 55–70 ms after stimulus onset (Wilson et al., 1983; Baseler and Sutter, 1997; Luck et al., 1997; Vanni et al., 2001; Foxe and Simpson, 2002; Di Russo et al., 2003; Boehler et al., 2008). However, it is worthy to note that the magnocellular and parvocellular pathways have different response latencies (Maunsell and Gibson, 1992; Schmolesky et al., 1998), with the magnocellular pathway responding earlier and maintaining approximately 10–15 ms advantage over the parvocellular pathway across areas in the early visual cortex (Schmolesky et al., 1998). From V1 information travels to temporal, parietal, and frontal cortices via feedforward connections in the ventral and dorsal streams (Ungerleider, 1985). Importantly, information not only propagates forward, but laterally and backwards; that is, hierarchically higher areas send signals to lower areas via feedback connections (DeYoe and Van Essen, 1988; Felleman and Van Essen, 1991; Van Essen et al., 1992; Merigan and Maunsell, 1993; Lamme and Roelfsema, 2000; Bullier, 2001) and information within an area is transformed via lateral neural connections (Felleman and Van Essen, 1991).

The role of feedback processes in vision as well as when and how they are initiated is still debated. Feedback amplifies and focuses activity of neurons in lower visual areas (Hupé et al., 1998, 2001). However, the function of such modulation is contested. Some argue that feedback is involved primarily in attentional modulation of the feedforward sweep (Macknik and Martinez-Conde, 2007), while others postulate that feedback modulates activity in early sensory areas based on expectations or to minimize prediction error (Rao and Ballard, 1999; Panichello et al., 2012). Several other models fall within a general “frame-and-fill” approach where feedback serves to fill in details of an initially established scene (Bullier, 2001; Hochstein and Ahissar, 2002; Bar, 2003; Ahissar and Hochstein, 2004; Bar et al., 2006; Chen et al., 2007; Kveraga et al., 2007; Ahissar et al., 2009; Peyrin et al., 2010). Regardless of its specific function, it is argued that this feedback activity is an essential component of an emergent visual awareness (Di Lollo et al., 2000; Enns and Di Lollo, 2000; Lamme, 2000, 2006a; Lamme and Roelfsema, 2000; Lamme et al., 2002; Tong, 2003; Breitmeyer, 2007; Fahrenfort et al., 2007, 2008). While these theories concur that feedback is necessary for awareness, we note that they should not be construed as arguing that feedback is sufficient for awareness. For example, feature integration can be modulated over hundreds of milliseconds even though the actual stimuli fail to reach awareness (Otto et al., 2006; Plomp et al., 2009; Scharnowski et al., 2009; Rüter et al., 2010), suggesting that while information is maintained in the visual system (presumably due to recurrent processing) additional factors determine whether it eventually becomes a conscious percept (e.g., see Herzog et al., 2007). In the context of visual awareness we explicitly define recurrent feedback as activity that encompasses recurrent processing within and among adjacent areas as well as reentrant activity from hierarchically higher to lower brain areas, the latter of which reside in the early visual cortex. Importantly, recurrent feedback occurs after and as a result of the initial feedforward signal. We will use “recurrent” and “feedback” as synonyms throughout.

It has been a challenge to establish the exact timing of these neural events because they are dependent on stimulus properties, species-specific neural architecture, and experimental procedures. For example, because magno and parvo cells exhibit distinct spatial frequency and contrast sensitivities (Kaplan and Shapley, 1986; DeYoe and Van Essen, 1988; Livingstone and Hubel, 1988; Van Essen et al., 1992; Schyns and Oliva, 1994; Sincich and Horton, 2005), the physical properties of the stimulus modulate signal arrival time in V1 and higher visual areas (Baseler and Sutter, 1997; Schmolesky et al., 1998; Alexander et al., 2005; Foxe et al., 2008). Nonetheless, some have estimated that activity occurring prior to 100 ms post-stimulus onset in the human brain corresponds to a feedforward signal, whereas feedback impacts activity in a later time period, 100 ms or more after stimulus onset (e.g., Fahrenfort et al., 2007; Boehler et al., 2008). However, if the definition of feedback encompasses any recurrent activity, as ours does, then such activity could also occur before 100 ms (e.g., Nowak et al., 1995; Foxe and Simpson, 2002). As will become clear in subsequent sections, it is difficult to pinpoint specifically when feedback might be playing a role in awareness, but by considering both visual masking and TMS studies together, we argue that the presence of both feedforward and feedback processes can be inferred.

It has been argued that some natural stimuli, such as real-world objects, animals, or scenes, can be perceived on the feedforward sweep; that is, observers can successfully identify such stimuli with presentation times that are too short to allow for feedback (Thorpe et al., 1996; VanRullen and Thorpe, 2001; Rousselet et al., 2002; VanRullen and Koch, 2003; VanRullen, 2007; Schmidt and Schmidt, 2009; Koivisto et al., 2014a). In these studies feedback is typically conceived although not explicitly defined as activity from higher to lower neural areas and, in particular, activity from frontal regions involved in decision making projecting to occipitotemporal areas. Others have argued that identification of such natural stimuli may rely on very fast feedback processes (Bar, 2003; Bar et al., 2006; Kveraga et al., 2007). However, we note that the concept of feedback processes as well as masking methods used in these ultra-rapid presentation paradigms do not strictly preclude recurrent processing and thus they cannot be taken as strong evidence against the idea that awareness requires feedback activity. We return to this issue in the next section.

Numerous theories and methods of visual masking exist, and the nuances of these models are beyond the scope of this review. Most models fit within the general framework of feedforward and feedback processing that we discuss in this paper. However, for completeness sake, we direct an interested reader to several excellent reviews that discuss alternative methods and theories of visual masking (Breitmeyer and Ganz, 1976; Breitmeyer, 1984; Bachmann, 1994; Francis, 1997, 2000; Di Lollo et al., 2000; Enns and Di Lollo, 2000; Breitmeyer and Öǧmen, 2000, 2006; Francis and Herzog, 2004; Macknik, 2006; Ansorge et al., 2007; Kouider and Dehaene, 2007; Hermens et al., 2008; Ghose et al., 2012; Goodhew et al., 2013; Bachmann and Francis, 2014).

## SUPPRESSING STIMULI FROM AWARENESS: BACKWARD MASKING

### VISUAL MASKING

Visual masking occurs when perception of one stimulus, a target, is reduced by the presence of a second stimulus, a mask. The strength of masking is quantified as the reduction in the visibility of some aspect of the target (e.g., its shape). In forward masking the mask stimulus precedes the to-be-discriminated target; in backward masking the mask follows the to-be-discriminated target. Metacontrast is a specific case of backward masking in which spatially contiguous but non-overlapping stimuli are used. Because the target and mask do not overlap spatially, and thus do not stimulate the same retinal cells, any interaction between the target, and the mask must occur primarily at the cortical level. In line with this idea, target visibility is strongly impaired under dichoptic presentation conditions in which target and mask stimuli are presented separately to each eye (Kolers and Rosner, 1960; Schiller and Smith, 1968; Weisstein, 1971; Breitmeyer and Kersey, 1981). Here, the visual signals from each stimulus can only interact at post-retinal levels.

**FIGURE 1:**
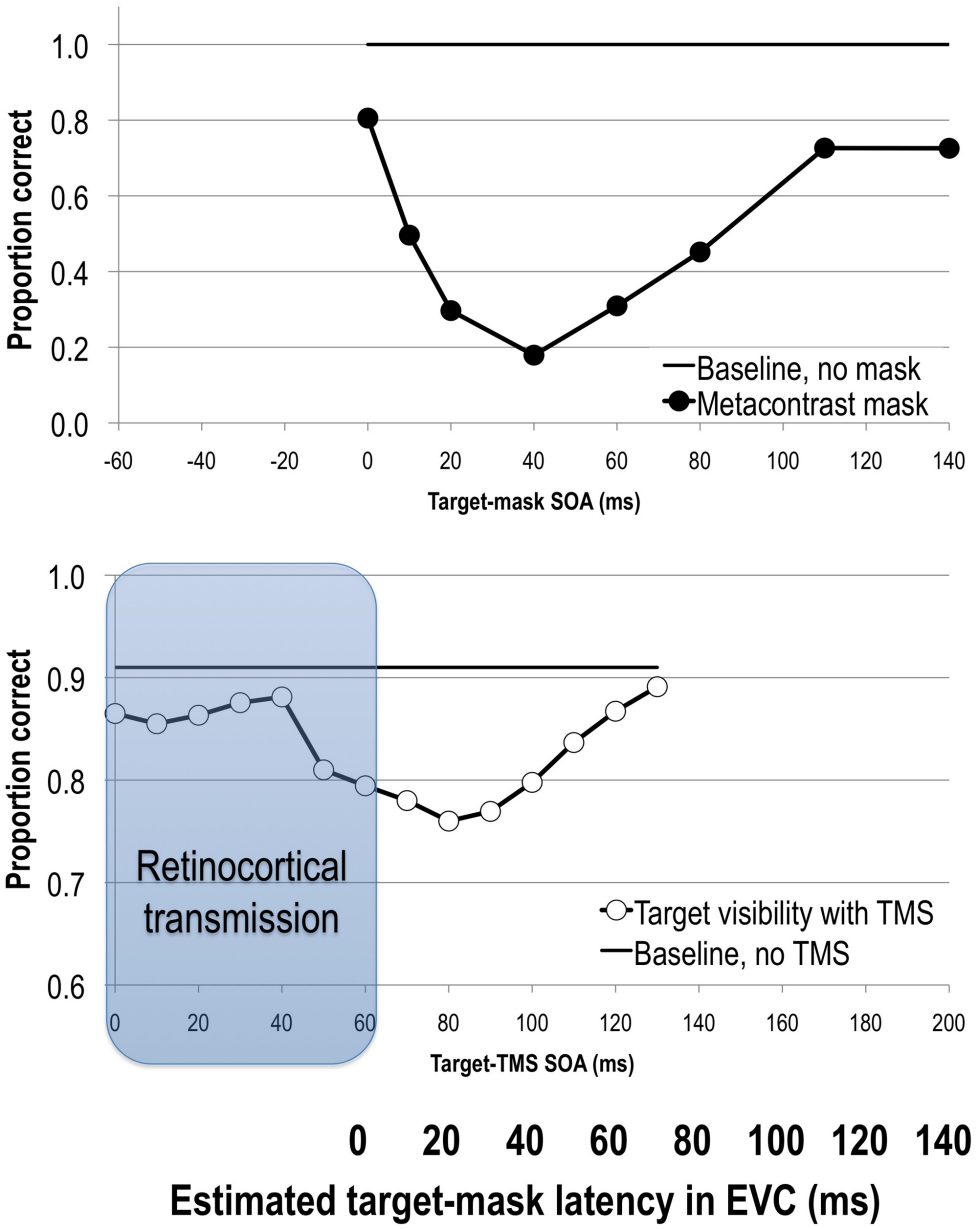
**Upper:** stimulus visibility in a metacontrast paradigm (adapted from Breitmeyer et al., 2006). **Lower**: stimulus visibility in a transcranial magnetic stimulation (TMS) paradigm (adapted from Tapia et al., 2014). When the average retinocortical transmission time of 60 ms (gray region) is taken into account, we can directly compare timing of neural events elicited by target and mask or TMS stimuli in EVC as indicated by the bolded horizontal axis. Both methods suppress stimulus visibility in comparable time windows, reflecting interference with the same visual process. See text for more details.

By varying the stimulus onset asynchrony (SOA) between the target and the mask one can track the change in the visibility of the target with respect to the onset of the mask. Metacontrast typically yields U-shaped target visibility functions (see **Figure 1**). The exact SOAs of optimal masking vary from study to study due to differences in experimental parameters and criterion the subject is asked to adapt (Kahneman, 1968; Breitmeyer and Öǧmen, 2006). In general, however, metacontrast masking produces the strongest suppression of target visibility at approximately a 50 ms SOA while preserving stimulus visibility at the earlier SOAs (e.g., Breitmeyer, 1978; Enns and Di Lollo, 1997; Breitmeyer et al., 2008; for reviews see Breitmeyer and Öǧmen, 2000, 2006; **Figure 1**). This pattern of results indicates that the strongest interaction between target and mask neural events occurs when they are separated by several tens of milliseconds rather than immediately.

The non-monotonic, U-shaped visibility of target function differs markedly from integration masking in which masking is most effective at an SOA of 0 ms and decreases in effectiveness monotonically with increasing SOAs (e.g., Hellige et al., 1977). This pattern of results suggests (nearly) immediate interactions between neural target and mask signals rather than after some delay. In other words, the very same SOAs that produce maximal integration masking (typically obtained when the target and mask spatially overlap) produce little or no masking during metacontrast (when target and mask do not spatially overlap). Whereas integration masking is largest when the mask is near in time to the feedforward signal of the target, metacontrast masking specifically circumvents that form of masking and instead affects some later processes beyond the initial feedforward signal.

Metacontrast and integration masking are also thought to differ in terms of where in the visual processing stream they occur. Integration masking is thought to occur when the target and mask stimulate common retinal and early cortical cells, resulting in an integration of target and mask signals starting at the earliest stages of processing (Weisstein, 1972; Breitmeyer, 1978; Hellige et al., 1979; Michaels and Turvey, 1979; Breitmeyer and Öǧmen, 2006). Metacontrast masking, on the other hand, is designed to minimize retinal integration and is thought to primarily reflect cortical interactions. It has been argued, however, that even these non-spatially overlapping stimuli may in fact be integrated (as opposed to interrupted, as it is sometimes described), although presumably at later levels of the visual system than the classic “integration masking” effects (Herzog and Koch, 2001; Otto et al., 2006; Plomp et al., 2009; Scharnowski et al., 2009; Rüter et al., 2010). In general, when masking methods are used to investigate awareness, care should be taken regarding the assumptions about the mechanism by which stimuli were suppressed from awareness. With this in mind, whenever relevant to our discussion we will only draw on evidence from masking studies with overlapping stimuli if cortical interactions between target and mask stimuli can be inferred (also see Breitmeyer and Öǧmen, 2006, for a further discussion on this point). Also, note that the latency differences between magnocellular and parvocellular channels are accommodated within the range of retinocortical transmission times discussed below, and thus we do not separate out the contributions of these pathways to visual masking effects and awareness.

### TRANSCRANIAL MAGNETIC STIMULATION

Another way in which to bypass sensory signal interactions in areas before V1 is to directly stimulate visual cortex with TMS. The rapid changes in the magnetic field generated by a TMS coil can induce electrical activity in the brain through the scalp and skull, thus non-invasively stimulating the brain area under the coil. In their seminal paper, Amassian et al. (1989) applied TMS to the occipital pole at varying SOAs relative to the stimulus onset. Reminiscent of metacontrast masking results, they found that occipital TMS reduced stimulus visibility when applied 80–100 ms after stimulus onset. Since then further research has confirmed that single-pulse TMS reduces target visibility in distinct temporal epochs when applied to the occipital pole, stimulation of which has since been shown to include V1, V2, and even V3 (Kammer et al., 2005b; McKeefry et al., 2009; Thielscher et al., 2010; Salminen-Vaparanta et al., 2012). This suppression of visibility effect has been more consistently reported as a window centered at a post-stimulus SOA of 100 ms (Amassian et al., 1989; Paulus et al., 1999; Corthout et al., 1999a,b, 2002; Heinen et al., 2005; Kammer et al., 2005b; Kammer, 2007a; Sack et al., 2009; Camprodon et al., 2010; de Graaf et al., 2011a, 2012a; Koivisto et al., 2011b; Railo and Koivisto, 2012; Emmanouil et al., 2013; Allen et al., 2014; Tapia et al., 2014; **Figure 1**). Several studies have also reported an additional and very late post-stimulus TMS effect at 200 to 280 ms after the onset of the stimulus (Allen et al., 2014), some in tasks that required animal categorization and relied on figure-ground segmentation processes (Heinen et al., 2005; Camprodon et al., 2010; Koivisto et al., 2011a).

Unlike in visual masking, where both target and mask enter the visual system via the retina, in TMS masking studies an externally induced TMS event applied directly over EVC affects an internal event in EVC that was initiated at the retina. Given that the retinocortical transmission time varies from 55 to 70 ms and averages around 60 ms (Wilson et al., 1983; Baseler and Sutter, 1997; Vanni et al., 2001; Foxe and Simpson, 2002; Boehler et al., 2008), any effect of TMS must first account for this retinocortical transmission time. For example, if we assume that it takes approximately 60 ms after the stimulus onset before TMS can directly interact with the incoming visual signal, then an effect of TMS on visibility at the 100 ms SOA would mean that TMS had its effect on the visual information approximately 40 ms after the sensory signal reached EVC.

After a retinocortical transmission adjustment is made, we can begin to compare SOAs in visual and TMS masking studies (see also Railo and Koivisto, 2012). **Figure 1** shows target visibility functions in metacontrast and post-stimulus TMS studies with the 60 ms adjustment. The 50 ms backward masking effect dovetails with post-stimulus TMS effects at an EVC-adjusted SOA of 40 ms (i.e., 100 ms post-stimulus onset). In other words, visual and TMS masking paradigms impair stimulus visibility at comparable time windows, suggesting that they affect the same visual process(es) that are critical for visual awareness (Breitmeyer et al., 2004a; Lamme, 2006b; Breitmeyer, 2007).

### NEURAL MECHANISMS

Data regarding the neural effects of backward masking is consistent with the idea that the mask interferes with feedback rather than an initial feedforward signal of the target. Single cell responses during backward masking have been recorded from a number of different primate brain areas involved in visual processing. In area V1 metacontrast masks suppress spike activity in a later time window (post-100 ms) while the early neural response is generally unaffected (Bridgeman, 1980; Macknik and Livingstone, 1998). Similarly, masking specifically disrupts signals responsible for differentiating figure from ground which occur in a later time window (post-100 ms), whereas it has no effect on the early signals (pre-100 ms) which reflect orientation differences of the elements comprising the figure-ground stimuli (Lamme et al., 2002). Because figure-ground segmentation depends on feedback from extrastriate areas to V1 (Lamme et al., 1998), these results suggest that the mask interferes with the feedback processing of the target. Furthermore, only the late, but not the early component of V1 responses correlates with behavioral reports of stimulus visibility, with higher neural activation observed for seen stimuli (Bridgeman, 1980; Lamme et al., 2000; Supèr et al., 2001).

Electroencephalography recordings with humans reveal similar findings to those of single-cell recording studies. Fahrenfort et al. (2007) presented texture-defined target stimuli and partially overlapping backward masks to human observers during a target detection task. In trials where the target was seen (i.e., not masked), three stages of visual processing indicated the presence of a figure (target) against a background. The first stage, which occurred during the first 110 ms after stimulus onset and was apparent in occipito-temporal electrode activity, was interpreted to reflect an initial feedforward activation of the visual system extending into the ventral stream. A second stage, which started at 110 ms after stimulus onset and was apparent in occipital electrodes, was interpreted as reflecting a reactivation of occipital regions due to recurrent feedback. A distinct third stage, which occurred around 200 to 300 ms and was apparent in occipito-temporal electrodes activity, was interpreted as reflecting an additional wave of recurrent activity. Importantly, the authors found that in trials where target was masked (i.e., not seen), both the second and third stages were no longer apparent; only the first stage of processing was unaffected by masking. In other words, the stages indicative of recurrent feedback were affected most, supporting the position that backward masks interrupt feedback processing of the target stimuli in the visual cortex. Together, single cell recording and human neuroimaging studies suggest that backward masking interrupts the later but not the initial neural response; these two stages of processing have been attributed to feedback and feedforward processing, respectively, (Fahrenfort et al., 2007, 2008; Boehler et al., 2008).

With this mechanism of masking in mind let us return briefly to studies that employ overlapping target and mask stimuli to make inferences about feedforward and feedback processes and their contribution to visual awareness. As discussed earlier, stimuli that overlap in space activate identical cells along the visual processing pathway allowing masking to occur due to sensory signal integration at pre-cortical levels. Because these studies typically employ pattern masks, successful discrimination of natural stimuli under ultra-rapid presentation conditions (i.e., target-to-mask SOAs of less than 100 ms) likely indicate poor integration masking rather than scene processing that does not require feedback; that is, fairly high target accuracy even at the shortest SOAs (Bacon-Macé et al., 2005; Walther et al., 2009; Loschky et al., 2010) may indicate that the masks used to interfere with natural scenes are simply in effectively integrated with the feedforward signal of the target. Indeed, both faces and scenes are most effectively masked by face and scene stimuli, respectively, (Loﬄer et al., 2005; Loschky et al., 2010), suggesting that other masks (e.g., noise) may be too simple or may not be effectively integrated with the rich feedforward signal from natural images. With these methodological considerations in mind we argue that data obtained using ultra-rapid presentation paradigms do not strictly preclude recurrent processing and thus they cannot be taken as strong evidence against the idea that awareness depends on feedback processes (see also Koivisto et al., 2014a). In fact, as mentioned earlier, some evidence exists to suggest that TMS over the occipital pole might interfere with feedback signals from scene stimuli in a 100 ms and later time window (Camprodon et al., 2010; Koivisto et al., 2011a).

The neural mechanisms behind TMS-induced reduction of stimulus visibility are less clear, due primarily to unknown variation in retinocortical transmission time. If we accept a retinocortical transmission time of 60 ms (Railo and Koivisto, 2012), then the timing of TMS-induced masking is comparable to that of backward visual masking. Thus, the classic post-stimulus TMS effect at 100 ms, like visual masking, could be attributed to interference with feedback processing in the EVC (Corthout et al., 1999a,b; Breitmeyer et al., 2004a; Lamme, 2006b; Breitmeyer, 2007; Allen et al., 2014). Even given some variability in retinocortical transmission time, it has been argued that such a late effect is unlikely to be due to TMS interference with the initial feedforward sweep (Celebrini et al., 1993; Merigan and Maunsell, 1993; Nowak et al., 1995; Schmolesky et al., 1998; Lamme and Roelfsema, 2000) and instead is likely due to interference with feedback processes (Breitmeyer et al., 2004a; Lamme, 2006b; Breitmeyer, 2007). The idea that feedback to EVC is required for visual awareness is supported by joint backward masking and TMS studies. Here in trials where target’s visibility is suppressed by a visual mask, if TMS is applied at around 100 ms after the onset of the mask, target stimulus visibility greatly increases while that of the mask is suppressed by TMS. Such unmasking of the target and masking of the mask by TMS has been taken as evidence of recurrent feedback processing in EVC (Amassian et al., 1993; Ro et al., 2003).

To directly test similarities and differences between metacontrast and TMS, Railo and Koivisto (2012) compared masking effects using identical target stimuli with the same group of participants. Both methods impaired subjective target visibility ratings in comparable time windows; the optimal masking SOA was 33 ms in metacontrast and 75 ms in TMS trials. Assuming a retinocortical transmission time of 60 ms, Railo and Koivisto (2012) suggest that TMS interference with visual processing occurs slightly earlier than with metacontrast masks. Interestingly, neither TMS nor metacontrast impaired subjects’ ability to simply detect the target in a 2-alternative forced choice location task. These data suggest that while feedback may be necessary for subjective reports of awareness, the intact feedforward signal may be sufficient to enable above chance detection in a forced-choice location task (VanRullen and Koch, 2003; VanRullen, 2007).

Although the timing of the TMS and visual masking effects obtained by Railo and Koivisto (2012) can be accommodated in the common framework of interference with feedback processes in EVC, the fact that the TMS effect occurs slightly earlier (depending on actual retinocortical transmission time) than visual masking raises an alternative view. It has been suggested that TMS in this time window may interrupt (some of the) feedforward in addition to feedback activity (Sack et al., 2009; Koivisto et al., 2011b; de Graaf et al., 2012a, 2014; Miyawaki et al., 2012; Railo and Koivisto, 2012). There are several reasons to consider this hypothesis. The range of SOAs that are lumped into the classical TMS suppression window not only vary from 60 to 140 ms but can extend over tens of milliseconds (Amassian et al., 1989; Corthout et al., 1999a,b, 2002; Kammer et al., 2005b; Sack et al., 2009; Camprodon et al., 2010; de Graaf et al., 2011b, 2012a; Koivisto et al., 2011a,b; Jacobs et al., 2012b; Railo and Koivisto, 2012; Allen et al., 2014; Tapia et al., 2014). This suggests that the earliest effects might be occurring as sensory information is just arriving in EVC and raises a possibility that TMS interferes with multiple visual processes in a wide window (Koivisto et al., 2011b; de Graaf et al., 2014).

The hypothesis that the 100 ms TMS suppression window may reflect interference with (some of the) feedforward in addition to (multiple recurrent) feedback processes (Camprodon et al., 2010; Koivisto et al., 2011b; de Graaf et al., 2012a, 2014; Miyawaki et al., 2012; Railo and Koivisto, 2012) is only now being considered in empirical investigations. Here, paradigms that allow differentiating between feedforward and feedback processes are especially useful. The framework of visual awareness we have adapted for this review specifically states that feedback is required for awareness, while feedforward processes are sufficient to “prime” selective motor responses (Lamme and Roelfsema, 2000; Breitmeyer, 2007). This view is supported by masked priming studies (Klotz and Wolff, 1995; Klotz and Neumann, 1999; Vorberg et al., 2003). Here, a visual masking paradigm is adapted to measure priming by varying the similarity between the target-prime and mask-probe. Responses to the mask-probe are faster when the two stimuli match on a to-be-discriminated feature (e.g., color) as compared to when they do not. Interestingly, the target-prime affects responses to the mask-probe both in trials when the target-prime is seen and when its visibility is suppressed by the subsequent mask-probe. We and others (e.g., Lamme and Roelfsema, 2000; Chen and Treisman, 2009; Tapia and Breitmeyer, 2011) have interpreted this pattern of results as indicating that the feedforward sweep of activity elicited by the target-prime is sufficient to produce priming even though the target-prime fails to reach awareness due to the interruption of its feedback processes by the mask-probe. Masked priming effects have been consistently reported for various stimuli in a metacontrast paradigm (e.g., Klotz and Neumann, 1999; Schmidt, 2002; Vorberg et al., 2003; Breitmeyer et al., 2004b, 2005, 2007; Enns and Oriet, 2007; Breitmeyer and Hanif, 2008; Kentridge et al., 2008; Ro et al., 2009; Schmidt and Schmidt, 2010; Tapia et al., 2010, 2011, 2013; Tapia and Breitmeyer, 2011).

If post-stimulus TMS over EVC at around 100 ms interrupts solely feedback processes, we would also expect similar (i.e., feedforward-supported) effects in TMS paradigms; that is, we would expect to obtain priming from a TMS-masked target. Alternatively, if post-stimulus TMS also interferes with (some of the) feedforward processes during this time window, performance on tasks that rely on feedforward activity should be impaired; that is, we would expect priming to be diminished when the target is suppressed by TMS. Only a handful of studies have investigated this hypothesis to date, but they all show that priming is either reduced (Sack et al., 2009; Railo et al., 2012) or entirely absent (Jacobs et al., 2012a; Persuh and Ro, 2013) at TMS SOAs ranging from 60 to 100 ms post-stimulus, suggesting that TMS in this range might be interfering with feedforward processes.

It is difficult to draw clear conclusions about feedforward and feedback processes from these studies not only because there are only a few of them, but also because the experimental procedures used differ among the studies. TMS stimulation parameters (e.g., intensity, shape of coil) should be systematically explored in the priming task as they have been shown to differentially affect performance in other paradigms (Beckers and Hömberg, 1991; Kammer et al., 2005a; Hallett, 2007; de Graaf et al., 2011a; Reichenbach et al., 2011). Additionally, differences between behavioral tasks should also be examined. For example, it is not known whether priming effects differ between paradigms in which TMS is applied either between presentation of target-prime and mask-probe (Persuh and Ro, 2013), during (Railo et al., 2012) or at the end of this stimulus sequence (Sack et al., 2009; Jacobs et al., 2012a; Railo et al., 2012). Moreover, assessing target-prime visibility during the same (e.g., Persuh and Ro, 2013) or separate sets of trials (e.g., Jacobs et al., 2012a) might also lead to different results (Lin and Murray, 2014). A combination of any of these factors might influence when and if priming with TMS is observed. Nonetheless, we believe that this line of inquiry will yield significant insights into feedforward and feedback contributions to visual awareness once these experimental procedures are fine-tuned.

In summary, although it has been previously argued that the 100 ms post-stimulus window reflects TMS interference with feedback processing in EVC, data from the newest studies suggest that the classical TMS window of suppression might in fact encompass both the feedforward and feedback processes, with lower SOAs tapping into the feedforward and later SOAs tapping into the feedback activity (Koivisto et al., 2011b; de Graaf et al., 2012a, 2014; Miyawaki et al., 2012). Hence, while there is ample evidence to suggest that metacontrast and TMS interfere with feedback processes, future research should inform the effects of TMS on feedforward activity as well as how and why this method of masking differs from that of metacontrast.

## SUPPRESSING STIMULI FROM AWARENESS: FORWARD MASKING

### VISUAL MASKING

**FIGURE 2:**
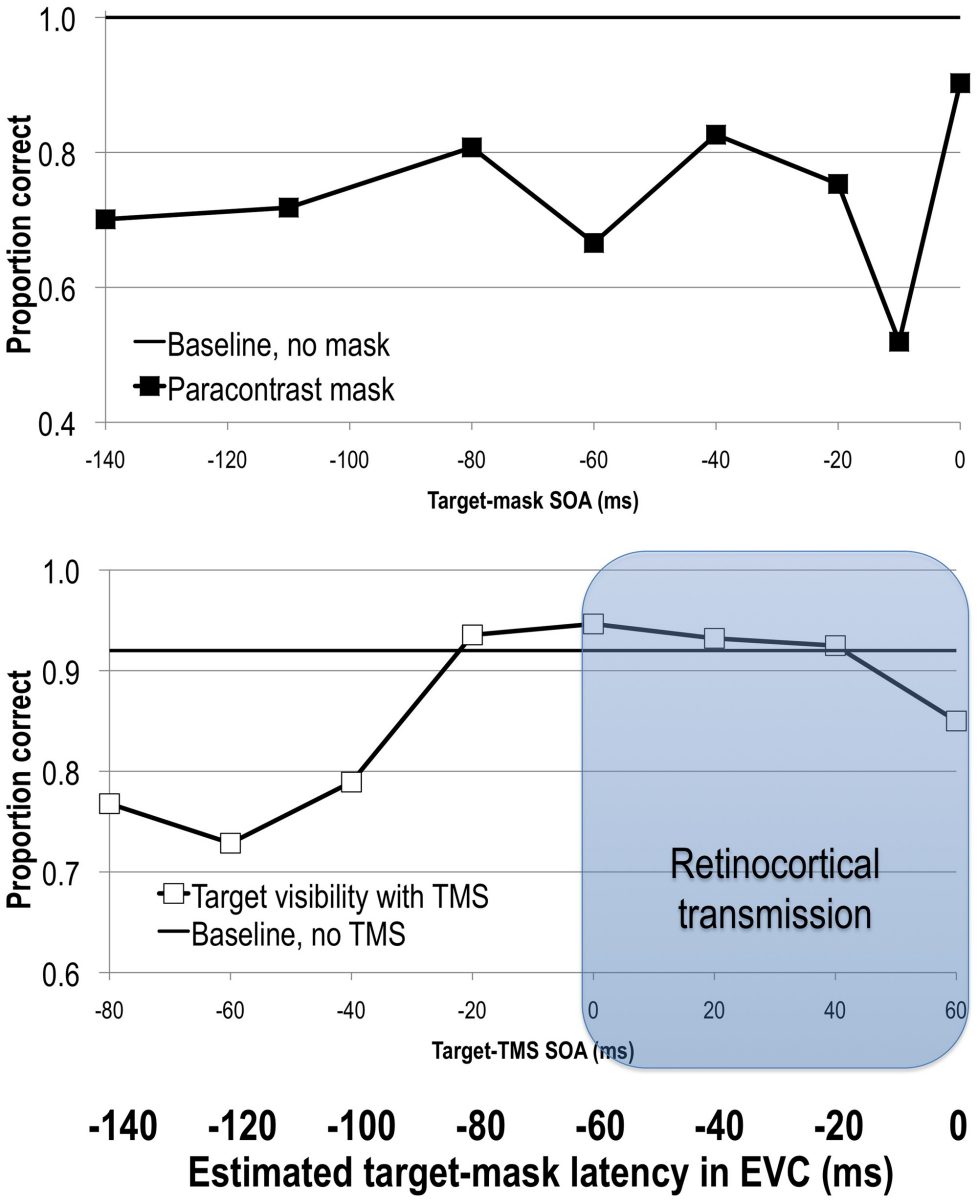
**Upper:** stimulus visibility in a paracontrast paradigm (adapted from Breitmeyer et al., 2006). **Lower**: stimulus visibility in a TMS paradigm (adapted from Jacobs et al., 2012b). When the average retinocortical transmission time of 60 ms is taken into account, visual signal reaches EVC at approximately 60 ms after TMS onset, as can be seen on the bolded horizontal axis depicting latency of elicited signals in EVC. See text for more details.

Given that feedforward processes precede and initiate feedback processes, it should also be possible to interfere with the initial feedforward sweep of activity and reduce stimulus visibility. Paracontrast is thought to be one such example. It is a specific case of forward masking that, like metacontrast masking, involves spatially non-overlapping target and mask stimuli. Unlike in metacontrast masking, here the mask stimulus precedes the target, and is thus thought to interfere with feedforward processing of the target. As in backward masking, varying the SOA between the target and the mask reveals distinct windows of visual suppression (**Figure 2**). In paracontrast strongest suppression of target visibility is obtained at SOAs around -170 to -100 ms and -10 ms (Cavonius and Reeves, 1983; Kaitz et al., 1985; Öǧmen et al., 2003; Breitmeyer et al., 2006).

### TRANSCRANIAL MAGNETIC STIMULATION

As in forward visual masking paradigms, TMS can also be applied before the onset of a visual stimulus. Pre-stimulus TMS impairs target visibility at a range of SOAs spanning from -80 to -10 ms (Corthout et al., 1999b, 2002, 2003; Laycock et al., 2007; de Graaf et al., 2011a; Jacobs et al., 2012b, 2014). Interestingly, pre-stimulus TMS at -20 ms shows retinotopically specific effects, that is reducing stimulus visibility only in specific locations, while TMS at -50 ms exhibits a global reduction of stimulus visibility regardless of where the stimulus was presented (Jacobs et al., 2014). Post-stimulus TMS effects shortly after the onset of the target (SOA effects at 0, 10, 20–60 ms; Beckers and Hömberg, 1991; Paulus et al., 1999; Corthout et al., 1999a,b, 2002, 2003) have also been reported (**Figure 2**).

### NEURAL MECHANISMS

Behavioral and especially neural effects of paracontrast are much less studied than those of metacontrast. This is likely due in part to the fact that paracontrast produces much weaker suppression than metacontrast (Alpern, 1953; Weisstein, 1972). Single-cell recordings in primate V1 reveal that paracontrast reduces the initial neural activity associated with the onset response of the target and slightly reduces subsequent excitatory after-discharge due to target offset (Macknik and Livingstone, 1998). Thus, it has been proposed that paracontrast masks interfere primarily with the incoming feedforward activity of the target (Breitmeyer and Öǧmen, 2006; Breitmeyer, 2007).

The neural consequence of the pre- and early post-stimulus TMS reduction of visibility is under question. Post-stimulus effects of SOAs up to 60 ms have been difficult to replicate consistently (Kammer, 2007a,b). Moreover, the SOAs of optimal suppression in pre-stimulus TMS studies, like in paracontrast, are quite varied (Beckers and Hömberg, 1991; Paulus et al., 1999; Corthout et al., 1999a,b, 2002, 2003; Laycock et al., 2007; de Graaf et al., 2011a; Jacobs et al., 2012b, 2014). In fact, some pre-stimulus TMS effects have been attributed primarily to eye-blink artifacts (Corthout et al., 1999b, 2011) raising questions as to whether it is a neural effect at all. More recent work, however, has shown that in trials where no eye-blinks occurred stimulus visibility was still significantly impaired with pre-stimulus TMS at -80 and -60 ms SOAs (Jacobs et al., 2012b, 2014).

Even with the masking effect localized to the cortex, the relationship between the pre-stimulus TMS effects and para- and metacontrast masking is still under debate. The early post-stimulus TMS effects at SOAs up to 60 ms have been explicitly attributed to TMS interruption of the feedforward sweep (Corthout et al., 1999a). However, given retinocortical transmission time, TMS applied earlier than that would presumably affect the visual cortex before the visual signal arrives there. Hence, it has been suggested that the pre-stimulus and early post stimulus TMS effects reflect changes in pre-stimulus brain activity, such as changes in alpha power or phase, rather than a direct effect on the evoked activity of the target (de Graaf et al., 2011a; Jacobs et al., 2012b, 2014). Such suggestions also raise the possibility that paracontrast masks also change brain states, such as those related to alpha power and phase. Moreover, although paracontrast and pre-stimulus and early post stimulus effects on stimulus visibility (when TMS SOAs have been adjusted for retinocortical delay) overlap in time, they do not align as nicely as with backward visual and TMS masks (see **Figure 2**). Of course, these conclusions critically depend on actual retinocortical transmission time. If one accepts that the backward masking effects of TMS and visual masks are comparable, then it is tempting to conclude that paracontrast and forward masking effects with TMS tap into the same visual mechanism of processing, this time interference with the feedforward signal (Corthout et al., 1999a,b; Breitmeyer et al., 2004a; Breitmeyer, 2007). As we have argued above, the post-stimulus TMS window likely reflects TMS interference with the incoming feedforward signal. If pre-stimulus TMS also affects the feedforward sweep, it may do this indirectly, by altering the brain state. Clearly, more research is needed on this issue.

## WHERE IS FEEDBACK COMING FROM?

Transcranial magnetic stimulation lends itself nicely to investigating temporal dynamics of visual processing because, unlike visual masks, it can be used to selectively interrupt activity in distinct visual areas (e.g., Pitcher et al., 2009). The timing of visual suppression effects when different regions are stimulated can inform us about the timing of feedforward and feedback processes between the stimulated areas. In a seminal study Pascual-Leone and Walsh (2001) provided evidence that feedback to V1 from V5/MT+ was necessary for the perception of motion. TMS over V5/MT+ produces the perception of moving spots of light (i.e., moving phosphenes). However, when TMS was applied to EVC between 5 and 45 ms after it was applied to V5/MT+, the perception of motion significantly decreased. Because TMS over EVC only interfered with motion perception subsequent to the V5/MT+ stimulation (motion suppression peaked at 25 ms post- V5/MT+ TMS), the result has been interpreted as TMS interference with feedback coming from V5/MT+ to EVC. Similar conclusions have been reached by others; that is, the perception of motion requires a feedforward and feedback exchange of activity between EVC and V5/MT+ (e.g., Silvanto et al., 2005a,b; Laycock et al., 2007; Koivisto et al., 2010).

This exchange of activity between EVC and extrastriate cortex is not limited to V5/MT+. Koivisto et al. (2011a) applied TMS to EVC and lateral occipital cortex (LOC) while participants categorized whether a briefly flashed image contained an animal or not, and rated their subjective visibility. TMS over EVC impaired categorization speed and subjective stimulus visibility ratings at SOAs from 90 to 210 ms after stimulus onset, whereas TMS over LOC affected categorization speed and subjective ratings only at the post-stimulus SOA of 150 ms. Such a pattern of data is consistent with the hypothesis that recurrent interactions between EVC and later regions, in this case LOC, are necessary for visual awareness, with the first suppression intervals produced by EVC and LOC stimulation corresponding to interference with feedforward signals and the later suppression window (at 210 ms) corresponding to interference with feedback from LOC into EVC.

TMS studies discussed thus far suggest that at least one source of feedback to EVC, which is needed for awareness, comes from extrastriate regions. However, a number of other brain areas have been implicated in awareness that might also act as a source of feedback to EVC. It is commonly argued that awareness arises due to interactions between fronto-parietal and occipito-temporal areas (e.g., for a review see Beck et al., 2001; Driver and Vuilleumier, 2001; Rees, 2001; Crick and Koch, 2003; Baars, 2005; Dehaene et al., 2006; Tononi and Koch, 2008). Studies with patients (e.g., unilateral neglect) and with TMS suggest that fronto-parietal regions are not incidental to awareness but critical to it (Driver and Vuilleumier, 2001; Turatto et al., 2004; Beck et al., 2006). Nonetheless, the exact dynamics of interactions between fronto-parietal and occipito-temporal regions are still under investigation.

One candidate for the source of feedback to EVC is the parietal lobe because of its abundant feedforward and feedback connections with occipito-temporal cortex (Felleman and Van Essen, 1991; Webster et al., 1994; Lewis and Van Essen, 2000). To test this possibility, Koivisto et al. (2014b) applied TMS to the EVC and the intraparietal sulcus (IPS). They found that shape visibility was impaired by EVC TMS at post-stimulus SOAs of 60, 90, and 120 ms, while TMS over IPS disrupted performance in the same task only at an SOA of 90 ms (Koivisto et al., 2014b). These results complement those from the authors’ earlier study where TMS over LOC impaired visibility in a relatively late time window (150 ms; Koivisto et al., 2011a), except that the IPS effect occurred earlier. In other words, both studies implicate recurrent interactions between EVC and higher-level areas, point to the importance of both the feedforward and feedback signals to awareness, and begin to address when and where that feedback is coming from.

Recently it was shown that phosphene sensations can be elicited with TMS applied over the parietal cortex, i.e., regions corresponding to the P3/P4 electrode sites (Marzi et al., 2009), and that these percepts are similar albeit less vivid than occipitally induced phosphenes (Fried et al., 2011; Mazzi et al., in press). Additionally, when TMS is applied to parietal areas that elicit phosphenes, occipital cortex exhibits activity 20 to 40 ms after parietal stimulation (Parks et al., 2013 and in preparation). The occipital activity is in line with the existence of feedback connections between parietal and occipito-temporal regions (Felleman and Van Essen, 1991; Webster et al., 1994; Lewis and Van Essen, 2000) as well as other data showing that TMS to parietal cortex can modulate activity in EVC (Ruff et al., 2008; Silvanto et al., 2009). What is not yet clear is whether the experience of parietal phosphenes (i.e., phenomenal awareness) requires the interplay of activity between parietal and EVC. Indeed, it is currently unknown whether occipital and parietal phosphenes arise from the same (Fried et al., 2011) or different neural mechanism given that parietally induced phosphenes have been reported in the blind visual field of two hemianopic patients (Mazzi et al., in press; see also Tapia et al., 2014).

The frontal lobes have also been implicated in visual awareness. The general “frame-and-fill” approach (Bullier, 2001; Chen et al., 2007) to visual processing posits that magnocellular channels project a rapid but coarse feedforward representation of the stimulus to higher cortical areas in the dorsal pathway and to the prefrontal cortex (Bullier, 2001; Peyrin et al., 2010; Tapia and Breitmeyer, 2011). Then, projections from these areas, activated by the initial magnocellular signals, potentiate or “frame” the processing along the slower ventral parvocellular pathway that carries the “fill” information, e.g., detailed form and color that are necessary for constructing a high-resolution representation of a visual object (Chen et al., 2007; Tapia and Breitmeyer, 2011; Breitmeyer, 2014). A specific version (Bar, 2003; Bar et al., 2006; Kveraga et al., 2007) of this “frame-and-fill” approach additionally posits that the prefrontal cortex projects directly to and modulates processing in the inferotemporal cortex (IT).

Finally, microstimulation of or TMS to frontal eye fields (FEF) have been shown to modulate neural responses in striate and extrastriate visual areas (Moore and Armstrong, 2003; Ruff et al., 2006, 2008; Silvanto et al., 2006; Taylor et al., 2007). The FEFs are considered a part of the dorsal attention network (Corbetta and Shulman, 2002). Given the link between attention and awareness and the connectivity of the FEF, these regions seem a reasonable candidate for awareness-related feedback to EVC. However, this feedback is yet to be shown to be critical to awareness. Given this complex neural network, the exact mechanism by which fronto-parietal regions generate feedback to EVC and contribute to visual awareness is yet to be determined and all of these candidates need to be probed further.

## THE NCCs OF VISUAL AWARENESS

Awareness is an emergent property of the brain and arises amidst other equally complex processes. Therefore, trying to pinpoint the neural correlate of consciousness (NCC) or more specifically of visual awareness may be misleading. For instance, there may be prerequisite conditions for the “true” NCC (or NCC-proper) to emerge as well as events that consistently arise as a result of awareness. The distinctions among these three NCCs have been discussed eloquently and in detail elsewhere (e.g., Aru et al., 2012; de Graaf et al., 2012b) and are inherently difficult to tease apart. Here we concentrate on the prerequisites of consciousness, or NCC-prerequisites, as some of the reported TMS effects are likely to fall into this category. Prerequisites include conditions set prior to the onset of a stimulus that by themselves cannot elicit a percept of that stimulus. Thus, as argued above, the pre-stimulus TMS effects (and possibly some of the early post stimulus effects) very likely achieve their effects by modulating brain states that influence how subsequent sensory information will be processed (Thut et al., 2006; Gilbert and Sigman, 2007; Mathewson et al., 2009; Summerfield and Egner, 2009).

Ongoing alpha oscillations (8-12 Hz) not only reflect a brain state that has been implicated in visual awareness, but they are also modulated by TMS. Visibility of a masked target in metacontrast fluctuates as a function of power and phase of occipito-temporal EEG alpha (Mathewson et al., 2009, 2010, 2012). Increased pre-stimulus alpha power has been associated with lower detection rates using a wide range of stimuli (e.g., Ergenoglu et al., 2004; Palva et al., 2005; Romei et al., 2008; van Dijk et al., 2008; Wyart and Tallon-Baudry, 2008; Busch et al., 2009; Mathewson et al., 2009). These changes in power have been linked to changes in attentional state (Worden et al., 2000; Thut et al., 2006; Mathewson et al., 2014). Others have argued that alpha power is an indication of the general level of excitability of the visual cortex, with high alpha power representing a general inhibition of ongoing processing (Klimesch et al., 2007; Mathewson et al., 2011). Consistent with this idea, Romei et al. (2008) have shown that identical TMS pulses over visual cortex are less likely to elicit visual phosphenes when alpha power over posterior cortical areas is high. Together these results suggest that the power and phase of alpha oscillations are indicative of a brain state that influences whether a subsequent stimulus will reach awareness.

Interestingly, even when alpha power is high there appear to be mechanisms that modulate awareness. Specifically, stimulus visibility varies as a function of alpha phase and is observed only under high alpha conditions (Mathewson et al., 2009, 2011, 2012). For instance, in metacontrast paradigm, depending on whether the target appears during a peak or trough in the alpha cycle it will be more or less likely to be detected (Mathewson et al., 2009, 2010, 2012). Similarly, alpha phase predicts whether or not participants will experience a TMS induced occipital phosphene (Dugué et al., 2011). Moreover, it is possible to induce alpha with either periodic visual stimuli (Mathewson et al., 2010, 2012; de Graaf et al., 2013; Spaak et al., 2014) or repetitive TMS (rTMS) over parietal cortex (Thut et al., 2011; Jaegle and Ro, 2014) and produce phase-dependent changes in stimulus detection performance. Romei et al. (2010) stimulated occipital and parietal cortex with rTMS at alpha (10 Hz), theta (5 Hz), and beta (20 Hz) frequency and found that stimulation only at the alpha frequency significantly correlated with stimulus visibility. Together these findings suggest that alpha is causally involved in shaping perception and, hence, both its phase and power reflect the brain state that can be labeled as NCC-prerequisites. Interestingly, activity in frontoparietal attention areas has been shown to correlate with the posterior alpha that predicts detection of visual stimuli (Mathewson et al., 2014), suggesting once again that pre-stimulus alpha may reflect attentional states.

**FIGURE 3:**
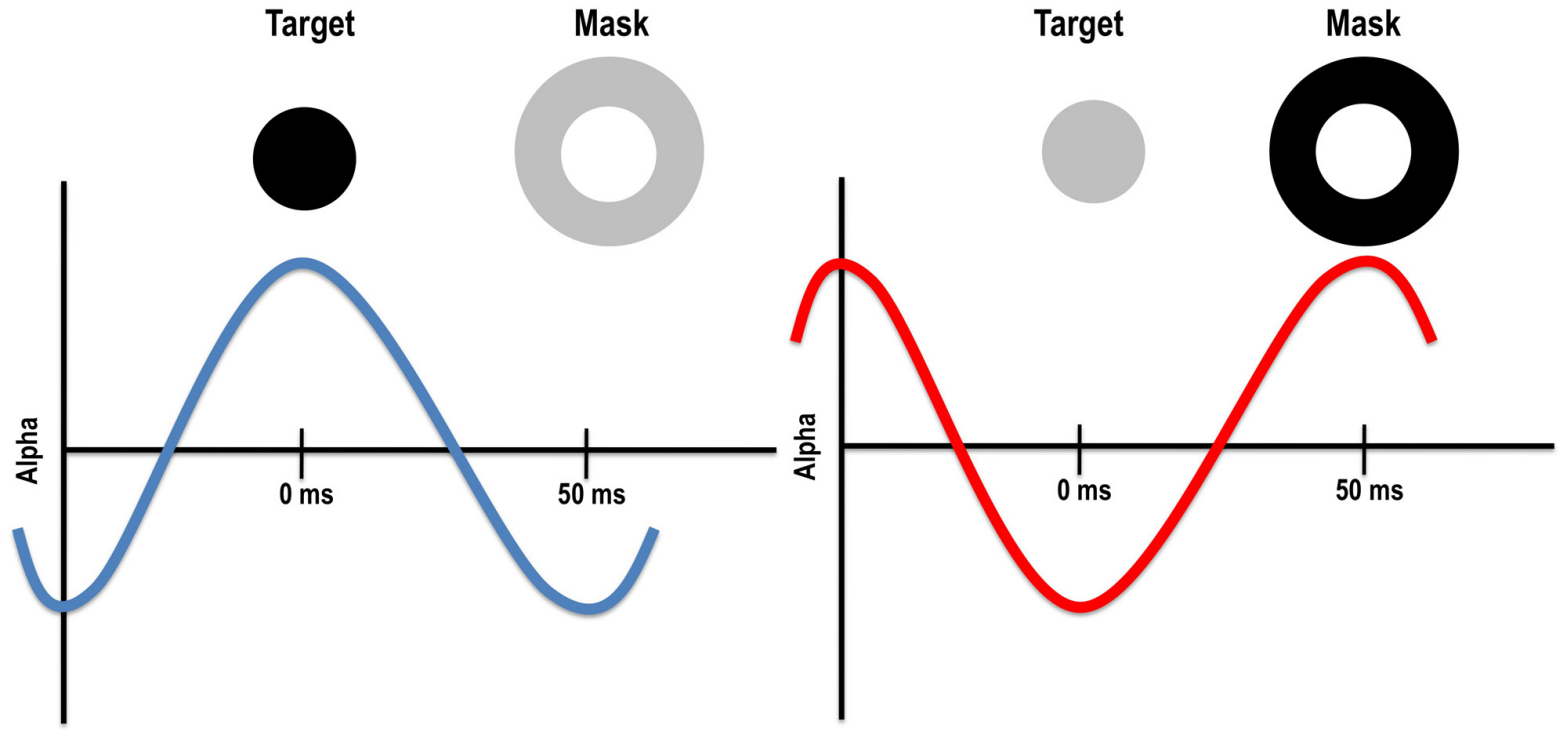
**Left:** target presented during the excitatory phase of the alpha cycle might be more visible because alpha enhances its visibility and/or because the mask falls within the inhibitory phase. **Right**: target presented during the inhibitory phase of the alpha cycle might be less visible because alpha reduces its visibility and/or because the mask falls within the excitatory phase

Given the timing of alpha and optimal backwards masking SOAs, it is possible that alpha oscillations impact both the target and mask. Specifically, optimal backward masking SOAs with visual and TMS masks (when retinocortical transmission time is accounted for) fall within the half cycle of alpha. Indeed, the timing is such that if the target appears in an inhibitory phase of alpha, the mask will fall in the excitatory phase, potentially increasing the chances that the feedforward signal from the mask will interfere with the target (see **Figure 3**). Of course, it is equally likely that the target appears during the excitatory phase of alpha, resulting in poorer masking and better detection of the target. Such a mechanism could explain why metacontrast masking rarely occurs on 100% of trials. Alpha phase alone cannot account for backward masking more generally, however, because alpha power is low when a study participant is fully engaged in the task. In this case, phase has little to no effect on stimulus visibility, yet metacontrast masking still occurs during low alpha power states, albeit at a reduced rate compared to trials occurring during high alpha power states (Mathewson et al., 2009). Further research is needed to understand the relationship between alpha power and phase, and paracontrast and pre-stimulus TMS effects. Because TMS pulses in a range of modalities have been shown to reset alpha (Mathewson et al., 2012; Romei et al., 2012), it is possible that a visual mask or TMS pulse impacts alpha and this in turn may explain some of reported pre-stimulus effects.

Finally, with regard to the NCCs we note that most data collected on consciousness are just that, correlates. We still do not know what conditions give rise to the experience of consciousness. However, we argue that the visual masking and TMS literature reviewed here suggests that feedforward and feedback signals are not only correlated with awareness, but necessary for it. We are not suggesting, however, that such signals are sufficient for awareness. We do not suppose, for instance, that any set of neurons wired in a recurrent fashion should result in awareness (Herzog et al., 2007). Indeed, feedback likely confers some other advantage such as allowing for integration of information (Oizumi et al., 2014) or resonance between top-down expectations and bottom-up input (Grossberg, 2013).

## CONCLUSION

To summarize, visual masking paradigms and TMS to EVC affect stimulus visibility in distinct time windows. Backward visual (metacontrast) and post-stimulus TMS masking effects have been thought to occur due to interference with feedback processes that are required for visual awareness, while forward visual (paracontrast) and pre-stimulus as well as early post-stimulus TMS effects have been proposed to reflect interference with the initial feedforward activity. Recent empirical evidence, however, suggests that the parallels between metacontrast and TMS masking might not be as straightforward as previously thought. While metacontrast occurs due to the mask interfering with feedback processes of the target, post-stimulus TMS possibly interrupts not only the feedback but also (some of the) feedforward processes. Additionally, forward masking (paracontrast) was thought to reflect interference with feedforward processing, but recent work looking at alpha oscillations raises the possibility that pre- and early post-stimulus TMS influence stimulus visibility by affecting the brain state prior to target onset. Future research should also inform the involvement of feedforward activity in post-stimulus TMS masking window and elucidate how and why this method of masking differs from that of metacontrast. Additionally, assessing the exact retinocortical transmission time for visual stimuli and specific time windows of suppression with TMS and visual masks should help disentangle feedforward and feedback contributions to visual processing in these paradigms. Finally, exploring the contributions of oscillatory alpha power and phase will help establish the necessary and sufficient conditions to visual awareness by parsing out prerequisites from NCC-proper.

## Conflict of Interest Statement

The authors declare that the research was conducted in the absence of any commercial or financial relationships that could be construed as a potential conflict of interest.
